# The Attitude of the Family Physician during an Epidemic of Poliomyelitis

**Published:** 1916-12

**Authors:** Irving M. Snow

**Affiliations:** Buffalo


### BUFFALO MEDICAL JOURNAL

Yearly Volume 72 DECEMBER, 1916 Number 5

### ORIGINAL ARTICLES

The right is reserved to decline papers not dealing with practical medical and surgical subjects, and such as might offend or fail to interest readers. Contributors are solely responsible for opinions, methods of expression and revision of proof.

---

### The Attitude of the Family Physician During an Epidemic of Poliomyelitis.

Dr. IRVING M. SNOW, Buffalo.

(Authorities: Wickmann, Flexner, Lovett. New York State Journal of Medicine, Sept. 1916.)

During the summer of 1916 the community of the Eastern and Middle States, was in a pitiable condition of terror, caused by an epidemic of poliomyelitis. The disease commenced in Brooklyn in May and June, spread through all of the metropolitan districts, invaded Long Island, Connecticut, New Jersey and New England, extending to Northern and Central New York.

The mortality and number of victims was the greatest in the history of the disease and there was a sad record of suffering, crippling and death.

Since 1907 our country has not been free from poliomyelitis, and high sanitary authorities predict that another epidemic may appear next year. Yet the history of the disease does not show an annual recurrence. Usually a neighborhood is free for several years. However, an attitude of preparedness is certainly advisable.

The state and local health officials are keenly on the alert, and new light has been thrown upon the epidemiology and bacteriology of poliomyelitis. With the health officials, the family physician is the main help of the community in arresting the spread of the plague, and both have fought skil-

*Read as part of Symposium on Infantile Paralysis at the Medical Association of Central New York, Buffalo, October 19, 1916.

fully and persistently. At present new and interesting duties are therefore thrust upon him; thus:—

I. The family physician should familiarize himself with the new facts of poliomyelitis, epidemiological and bacteriological, and also with the symptoms of both typical and atypical cases.

II. He should, without fear of the displeasure of his patients, promptly report and isolate all suspicious cases, and should personally use all the precautions of a surgical clinic to avoid spreading the disease himself.

III. He should co-operate with, and frequently confer with his health officer, so that the state, town, municipal authorities and practicing physicians may manoeuver like one man.

IV. He may also, by a courageous and tactful attitude, do much to tranquilize an infected locality, calling attention to the fact that the number of susceptible adults and children is always small.

Poliomyelitis shows most extraordinary variations in type and virulence from year to year. It may occur sporadically or in small isolated groups, or it may, during the warm season, increase by leaps and bounds leveling its victims by thousands. The disease seldom flows through a district like a stream; it jumps from one town to another miles away, or is sprinkled here and there through a city without reason or order.

It must be remembered that scattered, sporadic cases are peripheral to a central focus, and that the patient has been infected by a healthy or chronic carrier or by an unrecognized abortive case. The disease extends along the routes of ordinary travel,—land or water, railway or country road, and is distributed by human beings and their movements.

A few cases of poliomyelitis in New York in May and June, were the forerunners of a murderous epidemic in July and August. This year the apex of the plague was reached in August, in 1907 the maximum occurred in September.

Most cases develop between July and October. Yet the appearance of frost does not always kill the virus of poliomyelitis. Several winter epidemics have been reported. In the winter of 1913 poliomyelitis prevailed in Sweden and the apex of the disease was reached in April and May.

The winter incidence is not without importance in studying the epidemiology.

Regarding the fluctuations of the virulence of the germ,—Dr. Flexner finds an explanation in the experimental disease in monkeys. The poliomyelitis virus of man has a low infective power for monkeys; the disease has to be forced upon them. Through successive generations of monkeys the disease acquires increased virulence. After a number of generations of experimental simian disease it returns to its low infective power, corresponding to the rise and fall of an epidemic.

Thus:—in man after a long resting period the virus of infantile paralysis may again become actively infective, causing at first a few, then later numerous cases.

The germ infects the body sometimes through the digestive tract, more often through the mucosa of the throat and nose, reaching the central nervous system through the nasal lymphatics, and causes an acute hemorrhagic myelitis and a mild meningitis. The virus is contained in the secretions and is distributed in infectious droplets in sneezing, coughing, kissing, and by contaminated finger, handkerchiefs, or by the intestinal contents. Many epidemics have been traced to schools. The period of active infective power is greatest early in the disease, diminishes rapidly, and usually disappears at the end of five or six weeks. We would always think of recovered cases as chronic carriers and as a possible menace. Dr. Shepard relates several instances of direct and indirect transmission:

1. A public collector of accounts had a son ill with infantile paralysis. The case was not reported or isolated. Three cases of polio developed in houses where he visited.

2. Dr. Y. attended a case of poliomyelitis July 6th, using no precautionary measures. July 15th his infant daughter developed a right hemiplegia. Nothing further happened until fall, when he, as a school physician, examined 2000 children. Among them were several recovered cases of polio and children from previously infected families.

November 23rd his second daughter was attacked by poliomyelitis.

November 29th his third daughter sickened with the same disease.

Doctor Z. attended a case of poliomyelitis May 21st. A case developed in his family June 14th. In July a second abortive case occurred in his family. Dr. Z. was in intimate contact with his children while attending the first case.

June 24th an office patient of Dr. Z. sickened and died of polio.

Case 5: A child swung in a hammock with Dr. Z's first patient of ten weeks earlier, and later developed polio.

Flexner cites a case where the virus was carried in the throat for five months.

The mucosa of the throat and nose of a healthy person in intimate contact with infantile paralysis may become contaminated, and he may pass the disease to a susceptible child. The virus may lodge in the finger, clothes or handkerchief, and after several days infect the person,

To prevent the disease, nothing is better than syringing out the nose and throat with a saline solution.

To absolutely isolate all cases seems impossible, involving quarantine of all patients, contacts and attendants and abortive cases, closing the schools and exercising such surveillance over travel that practically means an arrest of commerce. Nevertheless, all foci of polio, large and small, should be watched and guarded.

Poliomyelitis attacks the rich and poor, sanitary and unsanitary, children and young adults.

Delicate and psychopathic children are no more easily affected than the robust. The age incidence, especially the susceptibility between two and five years, is very striking.

SYMPTOMS:

The disease commences with fever and malaise, headache, pain and tenderness in the back and limbs. Hyperesthesia or stiffness in the spine or neck and are very suggestive.

These symptoms may be difficult to localize in a fretful child. In an adult they simulate the myalgia of an influenza.

Vomiting and diarrhoea are often observed. The patient may have a sore throat or a head cold or a bronchitis. Prolonged drowsiness is very suspicious.

This may constitute the whole picture of the disease, but usually after one, two or three days some muscular groups grow weak and are easily fatigued, then loss of power appears, increases and attains its full extent in a few days, the patient being feverish, tender and prostrate. After some days or weeks the palsy recedes, owing to the subsidence of edema in the cord and absorption of inflammatory products. In the majority of cases a flaccid palsy remains.

Wickman's classification gives the clearest idea of the various clinical aspects of the disease:

1. Spinal paralysis.
2. Landry's paralysis.
3. Bulbar.
4. Encephalic.
5. Ataxic.
6. Meningeal.
7. Poly-neuritic.
8. Abortive.

A passing description of dangerous symptoms or atypical conditions may be useful.

The abdominal muscles are affected quite frequently, as shown by localized bulging of the abdominal walls, and difficulty in maintaining the erect position.

Paralysis of the bladder and retention of urine is often associated with paralysis of the legs. It is usually transitory like the fleeting muscular palsies.

Very dangerous are the paralyses of the respiratory muscles, the diaphragm and intercostals.

If the disphragm is paralyzed the abdomen is motionless.

If the intercostals are affected the chest does not move either in inspiration or expiration. Intercostal paralysis is bilateral; breathing is carried on by the diaphragm. If both intercostals and diaphragm are involved the patient quickly dies of asphyxia.

In recent epidemics of poliomyelitis the meningeal type has become common. It must be remembered that a constant lesion of Polio is a cell infiltration of the pia-mater and a mild meningitis at the beginning of an epidemic. Many deaths from Polio are reported as meningitis, often tuberculous meningitis.

The patient suffers from headache, fever, vomiting, retraction of the neck, opisthotonus, tenderness, rigidity and tremor of the limbs, strabismus and delirium. The symptoms may last several days and the patient may make a complete recovery, or more probably a paralysis of arm or leg are discovered as the symptoms of cerebral irritation subside.

The findings from lumbar puncture will be described by Dr. Sharp.

POLY-NEURITIC TYPE.

With the fever and prostration there may be extreme tenderness of the nerve trunks in arms and legs and spine. The patient shrieks if the bed is jarred or he is moved. Neuritic pains are nearly always followed by a paralysis of the tender limb. The pain is caused by a spinal meningitis.

Do not mistake the pain in the limbs for rheumatism. The joints are not swollen in Polio. Also remember that a baby with scurvy has weak, tender legs also a sore mouth, and is quickly relieved by fruit juices.

ABORTIVE CASES.

These are important to recognize. They are always seen with paralytic cases and are active factors in spreading the disease. The writer saw in one family a child with a flaccid palsy of the arm and three other children with fever, vomiting and diarrhoea. Between definitely abórtive cases with malaise and slight and fleeting palsies and frankly paralytic cases, several transitional forms may exist in the same family. A child may have fever, headache, pain, rigidity in the back and neck and stiffness in the limbs, pointing to a mild infection to the nervous system. Later it will show extreme prostration for weeks.

There may be passing symptoms of mild meningismas, rigidity of the neck and back.

Wickman describes four abortive types:

1. Cases running the course of a general infection.

2. Cases with meningeal irritation.
3. With limb tenderness—pseudo-influenza.
4. With gastro-intestinal symptoms, vomiting and diarrhoea.

A New York physician told the writer that during the 1916 epidemic seemingly abortive cases occasionally developed a fatal respiratory paralysis. Often abortive cases are not included in the collective investigations of an epidemic. They are forgotten or misinterpreted.

PROGNOSIS.

In 1916 Poliomyelitis showed itself in a new phase. The number of recovered cases was very large, but the mortality was abnormally high.

In the epidemics of 1907-1908 the death rate was from 5 to 7%. This year 26-28%, showing a striking increase in the virulence of the germ. Both the extent of residual palsies and percentage of fatalities was higher in older children and adults than in young children.

Wickman:—Mortality 1-11 years, 12%. 12-32 years, 27%.

Wickman found 12-18 months after the acute stage, 44% of children had completely recovered. 56% had a permanent paralysis.

In the American cases Lovett estimates only 10% had a serious disability like a paraplegia. Many of the permanent paralyses are slight; a weak leg or drop toe;—no more disabling in life work than imperfect eyes.

The outlook for restoring muscular function is much more favorable if the patients are not injured by premature walking movements or by electricity.

The writer visited the Willard-Parker Hospital in September and saw hundreds of convalescent children; most of them were rapidly improving. It was exceptional to see a badly crippled child.

The intensity of the initial symptoms is no index to the final result. Alarming early manifestations may usher in an abortive attack or a mild beginning may end in extensive palsy or a fatal respiratory paralysis.

TREATMENT.

No especial advance is recorded in treatment. Adrenalin has not proven especially useful. As to the serum of recovered cases,—convalescent serum,—Netter used the serum of a case whose acute stage occurred three years before and found that this serum in vitro destroyed the virus of epidemic poliomyelitis. Both Netter and Flexner proved that in the serum of recovered children antibodies exist.

Convalescent serum has been largely used in New York this summer by withdrawing 20 c.c. of spinal fluid and injecting 10 c.c. of serum into the spine. This remedy is enthusiastically advocated by some.

Lovett advises prolonged rest for five or six weeks to allow repair by absorption of the inflamed nerve center. He considers early massage, attempted movement and electrical treatment as injurious. With the disapearance of tenderness in six weeks active treatment may be commenced. A long stay in bed for children is both useless and undesirable.

Finally, if an epidemic appear next year, the health officials and family physician are centainly ready for it, armed with a full equipment of clinical experts, laboratories, hospitals, and a complete system of isolation and co-operation.

Alas! The experience of Sweden, scourged for years by frequent epidemics, shows that knowledge and preparedness do not always bring safety.

---

**Vision of the School Child.** F. Parke Lewis of Buffalo, N. Y., State Jour. of Med., Oct., quotes the following Pennsylvania statistics. In the report of 1914-15, in the fourth class districts, the number of pupils inspected amounted to 469,199. The tabulation of visual defects is as follows:

Tabulation of Defects.

Vision.

| | | | |
|---|---|---|---|
| Pupils having defective vision | | 83,748 | 17.8% |
| Right eye | 13,184 | | |
| Left eye | 14,750 | | |
| Both eyes | 55,814 | | |
| Pupils having other eye affections | | 5,512 | |
| Corneal scars | 1,717 | | |
| Blepharitis | 1,400 | | |
| Conjunctivitis, simplex | 1,237 | | |
| Conjunctivitis, follicularis | 7 | | |
| Iritis | 1 | | |
| Trachoma | 4 | | |
| Strabismus | 1,091 | | |
| Astigmatism (special tests not made | 55 | | |

---

**Gasoline Phlegmons.** Rene Dillenseger, These de Lyon 1916, Le Prog. Med., Sept. 5, reports two cases and refers to about 10 in literature. Diagnosis is made by the anthracoid appearance of the incision, multiplicity of foci, the gangrenous rather than purulent condition, the sterility, and the odor and chemic identification. As prophylaxis, he suggests a sentence of 5 years at hard work, under the legal charge of abandoning a post in face of the enemy, these phlegmons being usually self-produced to avoid or escape military service.

